# Lifestyle and Overweight Among Japanese Adolescents: The Toyama Birth Cohort Study

**DOI:** 10.2188/jea.JE20080095

**Published:** 2009-11-05

**Authors:** Yingchun Sun, Michikazu Sekine, Sadanobu Kagamimori

**Affiliations:** 1 Department of Welfare Promotion and Epidemiology, Graduate School of Medicine and Pharmaceutical Science for Research, University of Toyama, Toyama, Japan; 2 Shenyang Center for Disease Control and Prevention, Shenyang, China

**Keywords:** overweight, lifestyle factors, adolescents, Japan, Toyama birth cohort study

## Abstract

**Objective:**

To investigate the effects of lifestyle factors on overweight among Japanese adolescents.

**Methods:**

We studied 5753 junior high school students (2842 boys and 2911 girls) aged 12 to 13 years. The students were residents of Toyama prefecture, Japan and completed a questionnaire about their height, weight, and lifestyle factors, in June and July 2002. Subjects with a body-mass index (BMI) higher than age- and sex-specific cut-off points were defined as obese. Parental overweight was defined as a BMI of 25 or higher. Logistic regression analysis was used to examine associations between lifestyle factors and overweight.

**Results:**

Skipping breakfast, eating quickly, excessive eating, physical inactivity, and long hours of TV watching were positively and significantly associated with overweight in both sexes. There was a negative association between snacking and overweight in girls (*P* < 0.001); no such association was found in boys (*P* > 0.05). Nighttime snacking was negatively associated with overweight in boys and girls (*P* < 0.05). Extended video game playing (≥2 hours; OR = 2.00, *P* = 0.012) and short sleep duration (<7 hours; OR = 1.81, *P* = 0.004) were significantly associated with overweight in girls only. The respective risks of overweight that derived from the subjects’ fathers and mothers were 2.0 and 2.5 times, respectively, in boys and 1.9 and 3.0 times in girls.

**Conclusions:**

Parental overweight, skipping breakfast, eating quickly, excessive eating, long hours of TV watching, long hours of video game playing, physical inactivity, and short sleep duration were associated with adolescent overweight. Furthermore, there were significant negative associations between adolescent overweight and snacking in girls and nighttime snacking in both sexes.

## INTRODUCTION

The proportion of children and adolescents who are overweight and obese has increased dramatically throughout the world during the past few decades.^1^^–^^3^ In Japan, the prevalence of overweight in 12-year-old and 14-year-old adolescents increased from 9.2% and 8.6% to 14.9% and 11.2%, respectively, during the period from 1976 through 2000.^4^ Overweight and obesity have become an overwhelming global public health issue.^5^

Adolescence is a critical period in the development of overweight/obesity. Obesity in adolescents is associated with several serious medical complications, including type 2 diabetes, poor immune function, hypertension, metabolic disorders, decreased mobility, and sleep apnea, and may continue into adulthood.^6^^,^^7^ One study found that adolescent obesity increased the long-term (approximately 50-year) risks of adult morbidity and mortality, independent of adult obesity status.^8^ Another study found that 70% of obese 10- to 13-year-old children become obese adults.^9^ Accordingly, overweight/obesity has become one of the most important issues in adolescent and adult health.

In general, the causes of overweight and obesity among children and adolescents can be divided into genetic factors, such as parental obesity,^9^ and lifestyle factors, which include patterns of physical activity and diet.^10^^–^^12^ Apart from genetic factors, lifestyle is the most important factor in childhood and adolescent overweight and obesity. Some studies have shown that markers of an unhealthy lifestyle, such as inactivity, overeating, and short sleep duration, are related to overweight and obesity.^10^^–^^13^ However, findings are not consistent among studies, due to differences in variables such as age, sex, race, and region. Therefore, more studies are needed to confirm the putative associations between lifestyle and overweight/obesity, particularly so in Japan, where the number of studies of adolescent overweight is limited.^14^^–^^16^

In this study, we attempted to identify the associations between lifestyle factors and overweight in adolescence by surveying students aged 12 to 13 years who attended junior high schools in Toyama prefecture, Japan. In addition, differences between boys and girls were investigated.

## METHODS

### Subjects and procedure

The Toyama Birth Cohort Study is an ongoing follow-up study of lifestyle and health among all children born between 2 April 1989 and 1 April 1990 in Toyama Prefecture, Japan. By means of a questionnaire on lifestyle and a family history, the cohort children have been evaluated every 3 years since the inception of the study. The goal of the overall study is to clarify the effects of social, parental, and lifestyle factors on child health, and the details of this prospective study have been published elsewhere.^13^^,^^17^^,^^18^ The present study, which is the phase IV survey of the Toyama Birth Cohort Study, was conducted between June and July 2002. All the study subjects were aged 12 to 13 years and enrolled in the first academic year at one of 86 junior high schools in Toyama prefecture. In total, 10 453 students were included in the phase IV survey. A self-administered questionnaire was distributed throughout all junior high schools, excluding those for children with physical or mental disabilities. Of those who received a questionnaire, 9574 students (91.6%) responded. The following respondents were excluded from the analysis: those who did not answer one or more questions concerning age, gender, weight, height, the weight or height of parents, TV watching, physical activity, use of video games, eating habits, or sleeping habits. The remaining 5753 students (2842 boys and 2911 girls, 60.1% of the respondents) were the final study subjects.

The prefecture education authorities approved the content and ethical aspects of the study. Permission to participate in this follow-up study was obtained from all parents and students.

### Questionnaire

Each lifestyle factor item had 4 possible responses, as follows: (1) frequency of eating breakfast, snacking, and nighttime snacking: daily, almost daily, sometimes, and rarely; (2) eating speed: very fast, fast, normal, and slow; (3) eating volume: very large, large, normal, and small; (4) frequency of physical activity: very often, often, seldom, and never; (5) hours of TV watching per day: less than 1 hour, 1 to 2 hours, 2 to 3 hours, and at least 3 hours; (6) hours of video game playing per day: do not play video games, less than 1 hour, 1 to 2 hours, and at least 2 hours; (7) usual rising time: before 6:00 AM, 6:00 to 6:30 AM, 6:30 to 7:00 AM, and after 7:00 AM; (8) usual bedtime: before 10:00 PM, 10:00 to 11:00 PM, 11:00 to 12:00 PM, and after 12:00 PM; and (9) hours of sleep per day: less than 7 hours, 7 to 8 hours, 8 to 9 hours, and at least 9 hours. In April, the weight and height of students were measured and recorded to the nearest 0.1 kg and 0.1 cm by trained school nurses using a calibrated stadiometer and beam balance. The subjects were lightly clothed in bare feet, as per School Health Law. These measurements were recorded on the questionnaire. The heights and weights of parents were self-reported in the questionnaire. The self-reported heights and weights of adults have been proven to be reliable and can be used for younger adults.^19^^,^^20^

The body-mass index (BMI, weight in kilograms divided by the square of height in meters) of children and their parents were calculated. Childhood overweight was determined by age- and sex-specific cut-off points that were linked to an adult BMI of 25, as developed by the Childhood Obesity Working Group of the International Obesity Task Force (IOTF).^21^ The cut-off points were obtained by averaging data from 6 large nationally representative surveys, including 2 reference populations from Asia. The cut-off points for BMI in children were applicable to Japanese children. In the present study, the BMI cut-off points for overweight for children were 21.22 (age 12) and 21.91 (age 13) for boys, and 21.68 (age 12) and 22.58 (age 13) for girls. Parental overweight was defined as a BMI of 25 or higher, as per the criteria of the World Health Organization.^5^

### Statistical analysis

To better understand the associations between child lifestyle factors and overweight, we conducted a cross-sectional analysis. A longitudinal analysis of the associations of lifestyle and child overweight will be performed in a subsequent study.

The unpaired *t* test was used to compare differences by age, and the chi-squared test was used to evaluate the distributions of parental overweight and differences in lifestyle variables between boys and girls.

Logistic regression analysis was performed to evaluate the strength of the associations of parental overweight and lifestyle to adolescent overweight; boys and girls were analyzed separately. In model 1, logistic regression analysis was performed with adjustment for age only. In model 2, logistic regression analysis was performed with adjustment for age and parental overweight. In model 3, to control for potential confounding factors, logistic regression analysis was performed with adjustment for age, parental overweight, and other lifestyle variables. Odds ratios (OR) and 95% confidence intervals (95% CI) for overweight were calculated independently for each lifestyle variable, with dummy variables for the reference category.

Statistical analyses were conducted using SPSS 13.0. A 2-tailed *P* value of less than 0.05 was considered to indicate statistical significance.

## RESULTS

In the comparison between boys and girls there was no significant difference in age, paternal overweight, or frequency of nighttime snacking (Table 1). The prevalence of overweight among boys was 19.0%, and was significantly higher than that among girls (13.9%; χ^2^ = 27.9, *P* < 0.001). The distributions of all other lifestyle variables and maternal overweight significantly differed by sex. As compared to boys, girls more frequently reported eating breakfast irregularly, snacking, lower physical activity levels, eating slowly, watching TV for a longer time, rising later, and sleeping less. Boys tended to eat more quickly, be more physically active, and play video games longer per day.

**Table 1.** Distribution of variables among the study participants, by sex

Variables	Boys (*n* = 2842)	Girls (*n* = 2911)	*P* value
Age (years), mean (sd)	12.27 (0.45)	12.25 (0.44)	0.165^a^
Height (cm), mean (sd)	154.2 (8.0)	152.8 (5.7)	<0.001^a^
Weight (kg), mean (sd)	46.0 (10.2)	44.8 (8.0)	<0.001^a^
Overweight, *n* (%)			
​ Yes	541 (19.0)	404 (13.9)	<0.001^b^
​ No	2301 (81.0)	2507 (86.1)	
Paternal overweight, *n* (%)			
​ Yes	873 (30.7)	928 (31.9)	0.342^b^
​ No	1969 (69.3)	1983 (68.1)	
Maternal overweight, *n* (%)			
​ Yes	300 (10.6)	376 (12.9)	0.005^b^
​ No	2542 (89.4)	2535 (87.1)	
Breakfast, *n* (%)			
​ Daily	2558 (90.0)	2554 (87.7)	0.001^b^
​ Almost daily	176 (6.2)	259 (8.9)	
​ Sometimes	76 (2.7)	77 (2.6)	
​ Rarely	32 (1.1)	21 (0.7)	
Snacking, *n* (%)			
​ Daily	658 (23.2)	676 (23.2)	<0.001^b^
​ Almost daily	962 (33.8)	1183 (40.6)	
​ Sometimes	673 (23.7)	633 (21.7)	
​ Rarely	549 (19.3)	419 (14.4)	
Nighttime snacking, *n* (%)			
​ Daily	661 (23.3)	638 (21.9)	0.144^b^
​ Almost daily	252 (8.9)	286 (9.8)	
​ Sometimes	516 (18.2)	485 (16.7)	
​ Rarely	1413 (49.7)	1502 (51.6)	
Eating speed, *n* (%)			
​ Very fast	184 (6.5)	51 (1.8)	<0.001^b^
​ Fast	862 (30.3)	461 (15.8)	
​ Normal	1488 (52.4)	1883 (64.7)	
​ Slow	308 (10.8)	516 (17.7)	
Eating volume, *n* (%)			
​ Very large	171 (6.0)	52 (1.8)	<0.001^b^
​ Large	772 (27.2)	459 (15.8)	
​ Normal	1687 (59.4)	2086 (71.7)	
​ Small	212 (7.5)	314 (10.8)	
Physical activity, *n* (%)			
​ Very often	893 (31.4)	496 (17.0)	<0.001^b^
​ Often	1301 (45.8)	1220 (41.9)	
​ Seldom	578 (20.3)	1024 (35.2)	
​ Almost never	70 (2.5)	171 (5.9)	
TV watching (hours), *n* (%)			
​ <1	410 (14.4)	421 (14.5)	<0.001^b^
​ 1–2	1150 (40.5)	1063 (36.5)	
​ 2–3	799 (28.1)	772 (26.5)	
​ ≥3	483 (17.0)	655 (22.5)	
Video game playing (hours), *n* (%)			
​ 0	608 (21.4)	1681 (57.7)	<0.001^b^
​ <1	1208 (42.5)	840 (28.9)	
​ 1–2	789 (27.8)	291 (10.0)	
​ ≥2	237 (8.3)	99 (3.4)	
Rising time, *n* (%)			
​ Before 6:00 AM	220 (7.7)	167 (5.7)	<0.001^b^
​ 6:00–6:30 AM	905 (31.8)	962 (33.0)	
​ 6:30–7:00 AM	1204 (42.4)	1371 (47.1)	
​ After 7:00 AM	513 (18.1)	411 (14.1)	
Bedtime, *n* (%)			
​ Before 22:00 PM	716 (25.2)	396 (13.6)	<0.001^b^
​ 22:00–23:00 PM	1431 (50.4)	1404 (48.2)	
​ 23:00–0:00 PM	583 (20.5)	926 (31.8)	
​ After 0:00 PM	112 (3.9)	185 (6.4)	
Sleep duration (hours), *n* (%)			
​ <7	385 (13.5)	646 (22.2)	<0.001^b^
​ 7–8	1221 (43.0)	1376 (47.3)	
​ 8–9	989 (34.8)	751 (25.8)	
​ ≥9	247 (8.7)	138 (4.7)	

^a^*t*-test

^b^Pearson chi-square test

Tables 2 and 3 show the associations between lifestyle variables and overweight in Japanese junior high school students. Skipping breakfast, eating fast, excessive eating, physical inactivity, and long hours of TV watching were all positively and significantly associated with overweight in both boys and girls in all models. Although there was a negative association between snacking and overweight in girls (*P* < 0.001), no such association was found in boys (*P* > 0.05). Nighttime snacking was negatively associated with overweight in boys and girls in all models (*P* < 0.05). No significant association between either rising time or bedtime and overweight was found for either sex in the 3 models (data not shown). Although long hours of video game playing and short sleep duration were significantly associated with overweight in both sexes, the associations disappeared in boys after adjustment for age, parental overweight, and other lifestyle factors. We also found that parental overweight was strongly associated with overweight in their children. In boys, the risks of overweight derived from their fathers and mothers were 2.0 times and 2.5 times, respectively; the corresponding values in girls were 1.9 times and 3.0 times.

**Table 2.** Associations between lifestyles and overweight in boys aged 12–13 (*n* = 2842)

Variables	Not overweight (*n* = 2301) *n* (%)	Overweight (*n* = 541) *n* (%)	Model 1		Model 2		Model 3	
		
			OR (95%CI)	*P* value	OR (95%CI)	*P* value	OR (95%CI)	*P* value
Father								
​ Overweight	631 (27.4)	242 (44.7)	2.10 (1.73–2.55)	<0.001	—	—	1.97 (1.59–2.45)	<0.001
​ Not overweight	1670 (72.6)	299 (55.3)	1.00				1.00	
Mother								
​ Overweight	190 (8.3)	110 (20.3)	2.78 (2.14–3.61)	<0.001	—	—	2.49 (1.85–3.34)	<0.001
​ Not overweight	2111 (91.7)	431 (79.7)	1.00				1.00	
Breakfast								
​ Daily	2091 (90.9)	467 (86.3)	1.00		1.00		1.00	
​ Almost daily	131 (5.7)	45 (8.3)	1.53 (1.08–2.18)	0.018	1.50 (1.04–2.16)	0.030	1.85 (1.23–2.79)	0.003
​ Sometimes	57 (2.5)	19 (3.5)	1.50 (0.89–2.55)	0.130	1.51 (0.88–2.60)	0.135	1.49 (0.80–2.77)	0.206
​ Rarely	22 (1.0)	10 (1.8)	2.02 (0.95–4.30)	0.068	1.97 (0.90–4.30)	0.090	2.59 (1.05–6.40)	0.039
Snacking								
​ Daily	543 (23.6)	115 (21.3)	0.89 (0.66–1.19)	0.420	0.86 (0.64–1.16)	0.338	0.80 (0.57–1.12)	0.186
​ Almost daily	800 (34.8)	162 (29.9)	0.85 (0.65–1.12)	0.250	0.83 (0.63–1.10)	0.188	0.82 (0.60–1.12)	0.212
​ Sometimes	515 (22.4)	158 (29.2)	1.29 (0.98–1.70)	0.070	1.22 (0.92–1.62)	0.165	1.14 (0.83–1.56)	0.427
​ Rarely	443 (19.3)	106 (19.6)	1.00		1.00		1.00	
Nighttime snacking								
​ Daily	555 (24.1)	106 (19.6)	0.72 (0.56–0.92)	0.008	0.70 (0.55–0.90)	0.006	0.64 (0.49–0.85)	0.002
​ Almost daily	213 (9.3)	39 (7.2)	0.69 (0.48–0.99)	0.044	0.68 (0.47–0.99)	0.043	0.66 (0.44–0.99)	0.042
​ Sometimes	418 (18.2)	98 (18.1)	0.88 (0.68–1.14)	0.334	0.93 (0.71–1.20)	0.563	0.88 (0.66–1.18)	0.408
​ Rarely	1115 (48.5)	298 (55.1)	1.00		1.00		1.00	
Eating speed								
​ Very fast	113 (4.9)	71 (13.1)	10.31 (5.88–18.09)	<0.001	10.44 (5.91–18.45)	<0.001	4.16 (2.22–7.82)	<0.001
​ Fast	622 (27.0)	240 (44.4)	6.32 (3.84–10.42)	<0.001	5.96 (3.60–9.88)	<0.001	3.33 (1.94–5.72)	<0.001
​ Normal	1276 (55.5)	212 (39.2)	2.71 (1.64–4.45)	<0.001	2.68 (1.62–4.42)	<0.001	2.32 (1.36–3.95)	0.002
​ Slow	290 (12.6)	18 (3.3)	1.00		1.00		1.00	
Eating volume								
​ Very large	92 (4.0)	79 (14.6)	22.66 (10.51–48.88)	<0.001	21.11 (9.72–45.84)	<0.001	15.7 (6.93–35.83)	<0.001
​ Large	510 (22.2)	262 (48.4)	13.61 (6.60–28.03)	<0.001	12.74 (6.15–26.35)	<0.001	9.54 (4.50–20.23)	<0.001
​ Normal	1495 (65.0)	192 (35.5)	3.32 (1.61–6.85)	0.001	3.22 (1.56–6.65)	0.002	2.65 (1.26–5.57)	0.010
​ Small	204 (8.9)	8 (1.5)	1.00		1.00		1.00	
Physical activity								
​ Very often	792 (34.4)	101 (18.7)	1.00		1.00		1.00	
​ Often	1035 (45.0)	266 (49.2)	2.00 (1.56–2.56)	<0.001	2.00 (1.55–2.57)	<0.001	2.23 (1.70–2.94)	<0.001
​ Seldom	429 (18.6)	149 (27.5)	2.71 (2.05–3.58)	<0.001	2.65 (1.99–3.52)	<0.001	2.82 (2.05–3.87)	<0.001
​ Never	45 (2.0)	25 (4.6)	4.30 (2.53–7.32)	<0.001	4.28 (2.48–7.38)	<0.001	4.38 (2.34–8.19)	<0.001
TV watching (hours)								
​ <1	349 (15.2)	61 (11.3)	1.00		1.00		1.00	
​ 1–2	951 (41.3)	199 (36.8)	1.21 (0.88–1.65)	0.241	1.20 (0.88–1.66)	0.251	1.28 (0.90–1.82)	0.173
​ 2–3	644 (28.0)	155 (28.7)	1.38 (1.00–1.91)	0.049	1.40 (1.00–1.94)	0.047	1.29 (0.89–1.87)	0.173
​ ≥3	357 (15.5)	126 (23.3)	2.06 (1.47–2.90)	<0.001	1.96 (1.38–2.77)	<0.001	1.79 (1.21–2.67)	0.004
Video game playing (hours)								
​ 0	508 (22.1)	100 (18.5)	1.00		1.00		1.00	
​ <1	1002 (43.5)	206 (38.1)	1.04 (0.80–1.35)	0.757	1.03 (0.79–1.34)	0.831	0.88 (0.66–1.18)	0.402
​ 1–2	611 (26.6)	178 (32.9)	1.47 (1.12–1.93)	0.005	1.45 (1.10–1.91)	0.009	1.12 (0.82–1.53)	0.469
​ ≥2	180 (7.8)	57 (10.5)	1.62 (1.12–2.33)	0.010	1.50 (1.03–2.20)	0.034	0.91 (0.59–1.41)	0.675
Sleep duration (hours)								
​ <7	291 (12.6)	94 (17.4)	1.50 (1.13–1.99)	0.006	1.42 (1.06–1.90)	0.019	1.21 (0.82–1.77)	0.333
​ 7–8	991 (43.1)	230 (42.5)	1.08 (0.87–1.34)	0.513	1.04 (0.83–1.30)	0.744	0.97 (0.75–1.26)	0.819
​ 8–9	812 (35.3)	177 (32.7)	1.00		1.00		1.00	
​ ≥9	207 (9.0)	40 (7.4)	0.88 (0.61–1.29)	0.521	0.90 (0.61–1.32)	0.584	0.71 (0.46–1.10)	0.126

OR: odds ratio; 95% CI: 95% confidence interval

Model 1: ORs were adjusted for age.

Model 2: ORs were adjusted for age, paternal overweight, and maternal overweight.

Model 3: ORs were adjusted for age, paternal overweight, maternal overweight, and other lifestyle variables.

**Table 3.** Associations between lifestyles and overweight in girls aged 12–13 (*n* = 2911)

Variables	Not overweight (*n* = 2507) *n* (%)	Overweight (*n* = 404) *n* (%)	Model 1		Model 2		Model 3	
		
			OR (95%CI)	*P* value	OR (95%CI)	*P* value	OR (95%CI)	*P* value
Father								
​ Overweight	746 (29.8)	182 (45.0)	1.84 (1.49–2.31)	<0.001	—	—	1.89 (1.50–2.38)	<0.001
​ Not overweight	1761 (70.2)	222 (55.0)	1.00				1.00	
Mother								
​ Overweight	260 (10.4)	116 (28.7)	3.39 (2.63–4.37)	<0.001	—	—	3.03 (2.30–3.98)	<0.001
​ Not overweight	2247 (89.6)	288 (71.3)	1.00				1.00	
Breakfast								
​ Daily	2219 (88.5)	335 (82.9)	1.00		1.00		1.00	
​ Almost daily	216 (8.6)	43 (10.6)	1.32 (0.93–1.86)	0.119	1.32 (0.92–1.88)	0.133	1.19 (0.81–1.76)	0.373
​ Sometimes	60 (2.4)	17 (4.2)	1.88 (1.08–3.26)	0.025	1.96 (1.11–3.45)	0.020	1.76 (0.95–3.26)	0.073
​ Rarely	12 (0.5)	9 (2.2)	4.96 (2.08–11.87)	<0.001	5.14 (2.10–12.57)	<0.001	7.93 (2.79–22.53)	<0.001
Snacking								
​ Daily	618 (24.7)	58 (14.4)	0.37 (0.26–0.53)	<0.001	0.38 (0.26–0.55)	<0.001	0.31 (0.21–0.47)	<0.001
​ Almost daily	1049 (41.8)	134 (33.2)	0.50 (0.37–0.68)	<0.001	0.50 (0.37–0.689)	<0.001	0.45 (0.32–0.63)	<0.001
​ Sometimes	506 (20.2)	127 (31.4)	0.98 (0.72–1.34)	0.909	0.97 (0.70–1.33)	0.839	0.93 (0.66–1.30)	0.667
​ Rarely	334 (13.3)	85 (21.0)	1.00		1.00		1.00	
Nighttime snacking								
​ Daily	559 (22.3)	79 (19.6)	0.76 (0.58–1.00)	0.050	0.80 (0.61–1.06)	0.121	0.88 (0.65–1.19)	0.401
​ Almost daily	253 (10.1)	33 (8.2)	0.70 (0.48–1.04)	0.077	0.71 (0.47–1.05)	0.085	0.68 (0.44–1.04)	0.074
​ Sometimes	428 (17.1)	57 (14.1)	0.72 (0.53–0.98)	0.035	0.77 (0.56–1.06)	0.105	0.70 (0.50–0.98)	0.038
​ Rarely	1267 (50.5)	235 (58.2)	1.00		1.00		1.00	
Eating speed								
​ Very fast	41 (1.6)	10 (2.5)	3.06 (1.42–6.58)	0.004	3.11 (1.42–6.77)	0.004	2.92 (1.23–6.91)	0.015
​ Fast	354 (14.1)	107 (26.5)	3.82 (2.58–5.68)	<0.001	3.61 (2.41–5.40)	<0.001	2.55 (1.65–3.96)	<0.001
​ Normal	1634 (65.2)	249 (61.6)	1.92 (1.34–2.74)	<0.001	1.79 (1.24–2.56)	0.002	1.69 (1.15–2.47)	0.007
​ Slow	478 (19.1)	38 (9.4)	1.00		1.00		1.00	
Eating volume								
​ Very large	42 (1.7)	10 (2.5)	4.50 (1.92–10.57)	0.001	4.46 (1.87–10.66)	0.001	4.26 (1.63–11.13)	0.003
​ Large	350 (14.0)	109 (27.0)	5.86 (3.39–10.13)	<0.001	5.56 (3.19–9.68)	<0.001	5.47 (3.01–9.94)	<0.001
​ Normal	1817 (72.5)	269 (66.6)	2.77 (1.65–4.66)	<0.001	2.57 (1.52–4.34)	<0.001	2.70 (1.55–4.70)	<0.001
​ Small	298 (11.9)	16 (4.0)	1.00		1.00		1.00	
Physical activity								
​ Very often	458 (18.3)	38 (9.4)	1.00		1.00		1.00	
​ Often	1058 (42.2)	162 (40.1)	1.85 (1.28–2.67)	0.001	1.78 (1.22–2.59)	0.003	1.88 (1.27–2.78)	0.002
​ Seldom	855 (34.1)	169 (41.8)	2.39 (1.65–3.45)	<0.001	2.21 (1.51–3.21)	<0.001	2.35 (1.58–3.48)	<0.001
​ Never	136 (5.4)	35 (8.7)	3.11 (1.89–5.12)	<0.001	2.88 (1.73–4.80)	<0.001	3.61 (2.10–6.20)	<0.001
TV watching (hours)								
​ <1	383 (15.3)	38 (9.4)	1.00		1.00		1.00	
​ 1–2	939 (37.5)	124 (30.7)	1.33 (0.91–1.95)	0.143	1.31 (0.88–1.93)	0.179	1.40 (0.93–2.11)	0.103
​ 2–3	655 (26.1)	117 (29.0)	1.80 (1.22–2.65)	0.003	1.80 (1.21–2.68)	0.003	1.95 (1.29–2.96)	0.002
​ ≥3	530 (21.1)	125 (30.9)	2.39 (1.62–3.51)	<0.001	2.34 (1.58–3.47)	<0.001	2.37 (1.55–3.62)	<0.001
Video game playing (hours)								
​ 0	1474 (58.8)	207 (51.2)	1.00		1.00		1.00	
​ <1	713 (28.4)	127 (31.4)	1.27 (1.00–1.61)	0.052	1.26 (0.99–1.61)	0.065	1.16 (0.90–1.51)	0.257
​ 1–2	246 (9.8)	45 (11.1)	1.30 (0.92–1.85)	0.138	1.32 (0.92–1.88)	0.133	1.07 (0.73–1.57)	0.726
​ ≥2	74 (3.0)	25 (6.2)	2.42 (1.50–3.90)	<0.001	2.41 (1.48–3.94)	<0.001	2.00 (1.17–3.43)	0.012
Sleep duration (hours)								
​ <7	537 (21.4)	109 (27.0)	1.62 (1.19–2.20)	0.002	1.63 (1.19–2.24)	0.002	1.81 (1.21–2.72)	0.004
​ 7–8	1188 (47.4)	188 (46.5)	1.26 (0.96–1.65)	0.103	1.33 (1.01–1.77)	0.045	1.37 (1.00–1.88)	0.049
​ 8–9	667 (26.6)	84 (20.8)	1.00		1.00		1.00	
​ ≥9	115 (4.6)	23 (5.7)	1.59 (0.96–2.62)	0.071	1.69 (1.01–2.83)	0.045	1.53 (0.87–2.69)	0.137

OR: odds ratio; 95% CI: 95% confidence interval

Model 1: ORs were adjusted for age.

Model 2: ORs were adjusted for age, paternal overweight, and maternal overweight.

Model 3: ORs were adjusted for age, paternal overweight, maternal overweight, and other lifestyle variables.

## DISCUSSION

Many studies have reported that parental obesity, irregular diet, sedentary behavior, and poor sleep are associated with childhood and adolescent obesity.^10^^,^^13^^,^^17^^,^^22^ Consistent with these studies, we found that parental overweight, skipping breakfast, eating quickly, excessive eating, long hours of TV watching, long hours of video game playing, physical inactivity, and, in girls, short sleep duration were associated with adolescent overweight. Furthermore, we also found significant negative associations between adolescent overweight and snacking in girls and nighttime snacking in both sexes.

Earlier studies reported that overweight/obese children are inclined to have breakfast less frequently than normal weight children, and that when they eat breakfast, overweight/obese children have lower energy intakes and consume fewer nutrients than do normal weight children.^23^^,^^24^ Among children, skipping breakfast appears to be associated with a higher intake of high calorie foods and lower intakes of protein, vitamins, and minerals.^25^ Also, adolescents who ate less cereal and milk had a higher percentage of body fat.^26^ Thus, it has been widely assumed that skipping breakfast may lead to excessive weight gain. Our data confirmed that among adolescents who skipped breakfast there was a statistically significant prevalence of overweight. We affirm that skipping breakfast is an important determinant of adolescent overweight/obesity. Conversely, eating breakfast has been shown to reduce dietary fat.^27^ Moreover, starting each day with breakfast helps meet the recommended dietary allowances for vitamins A, B-6, and D, calcium, magnesium, riboflavin, zinc, phosphorus, and iron.^25^^,^^28^ Furthermore, in our study, overweight girls were prevalent among breakfast skippers. It is possible that in an attempt to control their body weight, adolescent girls do not eat breakfast.

With regard to other dietary habits, we found that excessive eating and eating quickly were associated with adolescent overweight. One explanation for this may be that excessive eating and rapid eating increase the amount of food entering the body, which leads to excessive energy intake, disruption of the energy balance in the body, and, ultimately, weight gain. Snacking is often looked upon as a source of excess caloric intake that contributes to an undesirable weight profile, and some studies have supported this opinion.^29^^,^^30^ However, the association between snacking and body weight is not consistent across studies.^31^ A review conducted by Cees de Graaf ^32^ found that the regularity of snacking was the essential determinant of adiposity. Because snacks are generally consumed on a more irregular basis than meals, people do not compensate for the energy intake from snacks consumed on an irregular basis. However, these explanations do not explain the negative association between snacks (including nighttime snacks) and adolescent overweight that was observed in the present study. A potential reason for our result is that adolescent changes in body composition cause boys to rapidly reduce body fat mass and girls aged 12 to 13 years—who are at peak height velocity—to quickly gain height. The energy intake from snacks or nighttime snacks may not be accumulated as excess energy in the body, but is instead absorbed for the development of the body during the adolescent growth spurt. Another potential reason is that snacks may contain little energy: greater general knowledge of prevention of overweight and obesity, and girls’ preferences regarding body shape, may encourage children to select low calorie foods when they snack. In a short-term intervention study, some researchers demonstrated that low calorie snacks could reduce the risk of overweight and obesity. Waller et al^33^ suggested that, among overweight or obese nighttime snackers, eating cereal after the evening meal reduced post-dinner energy intake and total daily caloric intake, and resulted in weight loss. The results of the present study support this earlier finding. Taken as a whole, these results provide new insight into the prevention of overweight and obesity, ie, adiposity can also be prevented or reduced by changing the composition of snacks, not just by changing snacking behavior.

As noted above, sedentary lifestyle patterns in children and adolescents have been associated with obesity. It is believed that the increased use of information and communication technology—particularly watching television, playing video games, and using computers—significantly contributes to a sedentary lifestyle, and thus to the increasing prevalence of obesity.^34^^,^^35^ In our study, playing video games (in girls), watching TV, and limited physical activity were strongly associated with overweight; however, playing video games in boys showed no such association after adjustment for other lifestyle factors. One reasonable explanation for this is that boys offset sedentary behaviors with higher physical activity levels. Indeed, boys (31.4%) were more active than girls (17.0%) in this study. Another possible explanation is that boys prefer video games with shooting, fighting, sports, and driving, while girls prefer adventure video games,^36^ which may result in different energy expenditures.^37^

Some epidemiological investigations have demonstrated that short sleep duration is a strong predictor of overweight/obesity^13^^,^^38^^,^^39^ and that there is sex difference in the relation between sleep duration and overweight/obesity.^38^^,^^40^ In contrast with the findings of Eisenmann JC et al,^38^ we found that short sleep duration (<7 hours or 7–8 hours) was associated with overweight in girls, but not in boys. In contrast with our findings from phase II, when children in the Toyama Birth Cohort Study were aged 6 and 7 years,^13^ the present study found no significant positive association between short sleep duration and overweight in boys. We believe that the difference in the association between sleep duration and childhood overweight may be due to gender differences in the physiological mechanisms of puberty, particularly with regard to changes in body composition. During puberty, the growth rates and hormone secretion levels of males and females differ.^41^ Males rapidly increase muscle mass and reduce body fat mass, due to increases in testosterone, growth hormone, and insulin-like growth factor-1.^41^^,^^42^ Females, on the other hand, experience rapid increases in fat mass during puberty, which may be largely due to increases in estradiol.^42^^,^^43^ These sex-specific effects on the endocrine system and metabolism explain why sleep duration has a different impact on weight gain in boys and girls.

It should be noted that our study has several limitations. First, the use of a cross-sectional study design precludes determination of the temporal sequence of lifestyle and overweight. Second, this study focused on a relatively narrow age range (12–13 years) of adolescents in Toyama Prefecture, Japan, and may not be nationally representative. Third, our interpretations in this study are limited to junior high school students who completed the study survey. Moreover, because items regarding total calorie intake and soft drinks were not included in the present study, the associations between dietary habits and adolescent overweight should be interpreted cautiously. Some of the lifestyle questionnaire items may be considered vague, which may lead to nondifferential misclassifications. However, because nondifferential misclassifications generally dilute the magnitude of the associations of factors with outcomes, the true relationship of factors and outcomes may be stronger than the observed relationship. Nonetheless, the findings from this study contribute to the body of evidence indicating that adolescents continue to require interventions that target multiple aspects of diet, physical activity, and sedentary and sleep behaviors. Such interventions would be likely to increase the proportion of adolescents who meet recommended health guidelines.

## CONCLUSIONS

This study showed that parental overweight, skipping breakfast, eating quickly, excessive eating, long hours of TV watching, long hours of video game playing, physical inactivity, and short sleep duration were associated with adolescent overweight. Furthermore, significant negative associations were found between adolescent overweight and snacking among girls and nighttime snacking among both sexes.
